# Mechanical Strength and Broadband Transparency Improvement of Glass Wafers via Surface Nanostructures

**DOI:** 10.3390/s16060902

**Published:** 2016-06-17

**Authors:** Amarendra Kumar, Kunal Kashyap, Max T. Hou, J. Andrew Yeh

**Affiliations:** 1 Institute of Nanoengineering and Microsystems, National Tsing Hua University, No. 101, Section 2, Kuang-Fu Road, Hsinchu 30013, Taiwan; s9935584@m99.nthu.edu.tw (A.K.); s9835878@m98.nthu.edu.tw (K.K.); 2 Department of Mechanical Engineering, National United University, No.2, Lienda, Miaoli 36063, Taiwan; max@nuu.edu.tw; 3 Department of Power Mechanical Engineering, National Tsing Hua University, No. 101, Section 2, Kuang-Fu Road, Hsinchu 30013, Taiwan; 4 Instrument Technology Research Center, National Applied Research Laboratories, 20, R&D Road VI, Hsinchu Science Park, Hsinchu 30076, Taiwan

**Keywords:** surface nanostructure, bending strength, transparency, borosilicate glass

## Abstract

In this study, we mechanically strengthened a borosilicate glass wafer by doubling its bending strength and simultaneously enhancing its transparency using surface nanostructures for different applications including sensors, displays and panels. A fabrication method that combines dry and wet etching is used for surface nanostructure fabrication. Specifically, we improved the bending strength of plain borosilicate glass by 96% using these surface nanostructures on both sides. Besides bending strength improvement, a limited optical transmittance enhancement of 3% was also observed in the visible light wavelength region (400–800 nm). Both strength and transparency were improved by using surface nanostructures of 500 nm depth on both sides of the borosilicate glass without affecting its bulk properties or the glass manufacturing process. Moreover, we observed comparatively smaller fragments during the breaking of the nanostructured glass, which is indicative of strengthening. The range for the nanostructure depth is defined for different applications with which improvements of the strength and transparency of borosilicate glass substrate are obtained.

## 1. Introduction

Glass is frequently used in almost all branches of engineering because of its mechanical and optical properties. Glass is commonly used for optical pressure sensors, displays, photovoltaic (PV) and other optical systems because of its high transmittance and lightweight nature [1,2]. Glass substrates are also used in micro-machined mechanical devices for acceleration or vibration sensing applications and panels because of the low cost of glass substrates compare to that of silicon [3,4]. Furthermore, glass is frequently used for chemical sensors, chemical glassware and biomedical applications because of its chemical inertness and biocompatibility [5,6]. Its strength is one of the most desirable mechanical properties for all of the above applications [3,7,8] and, therefore, improving the strength of glass has been a crucial area of study for decades.

There is a long history of efforts to improve the strength of glass. Surface defects are a major cause of strength reduction because they increase the local stress to a value that is much higher than the applied stress around corners, holes, and scratches [9,10]. Several strength improvement techniques are available, e.g., the reduction of the size of defects using coatings, the reduction of the severity of the defects, the introduction of surface compression, among others [11]. Coating or lamination can improve the strength by reducing the defect severity of the glass but it leads to a shorter lifetime [12]. Surface compression techniques, such as the ion-exchange method, can be applied to alkali-containing glasses only [13,14]. Reinforcement of glass with short fibers not only improves its strength but also changes the bulk property and processes for manufacturing the glass substrates [15]. All of these strength improvement methods can strengthen the glass substrates, but these methods either have no effect on transparency or can worsen it.

Furthermore, broadband transmittance and antireflection properties are also highly desirable for glass substrates in optical sensors, PV panels, displays and other applications [16,17]. Moth-eye-like nanostructures [18] and antireflective coatings (ARCs) [19] are established modifications that result in glass substrates with low reflection and high transparency. Fresnel reflection occurs when light travels between transparent media [20] of differing refractive indices. This change in refractive indices causes a reflection from the front surface that degrades the optical performance and thus reduces the transmission. ARCs are applied to achieve better transparency by reducing the reflection from the surface. This method requires specific designs to achieve narrow wavelength windows as well as the use of a precise thickness of ARCs [21]. Biomimetic subwavelength structures (SWSs), inspired by moth and butterfly corneas [22,23], have a smaller dimension compared to that of the incident light wavelength. Therefore, these structures have attracted increasing interest as alternatives to ARCs. These SWSs suppress the undesired Fresnel reflection losses using subwavelength texturing at the air-glass boundary [24]. All of the above-mentioned methods for transparency improvement have been reported to improve transparency only and do not affect mechanical strength.

Given the above limitations, the glass industry requires a strengthening technique that can improve strength without affecting optical properties or altering the current manufacturing processes. Surface nanostructure can improve the silicon substrate strength by stress redistribution, which consequently suppresses crack initiation [25,26,27]. Crystalline materials show higher resistances to crack initiation because of lattice trapping, which depends on the atomic configuration and cleavage planes [28], but for an amorphous material crack initiation is related to the material composition [29,30,31] and, therefore, lattice trapping is not possible. The strength of the silicon sample is enhanced [27] and the effects of the strength-degrading defects are eliminated [25] using surface nanostructures, but the effects of surface nanostructures on the material strength of amorphous substrates are unknown. Therefore, the effects of surface nanostructures on the mechanical strength of amorphous substrate need to be studied. This study explores the application of surface nanostructures for both mechanical strength and optical transparency improvement of amorphous borosilicate glass substrates.

More specifically, we present our findings, which demonstrate the achievement of both higher strength and better transparency compared to plain glass via SWS fabrication. SWSs can be fabricated using a photolithography mask; however, the fabrication steps are complex [32,33]. Thermal dewetting is a relatively simple, less expensive, and more widely reproducible process compared to e-beam, laser interference and nanoimprint lithography processes, but thermal dewetting is normally a wafer-by-wafer process, and a high temperature is needed [34]. Coating of nanoparticles on glass substrate has a similar masking function but a simultaneous coating of nanoparticles on both sides of the substrate can be difficult [35,36]. SWSs for the current study were fabricated using a combination of wet and dry etching techniques. This fabrication method is suitable for producing large substrates and has mass production capabilities. A single-side nanostructured substrate for display and PV applications to prevent particle entrapping between nanostructures can also be fabricated using this fabrication method. The optimum range of the nanostructure depth for both strength and transparency improvement of borosilicate glass substrate is also discussed for practical applications.

## 2. Materials and Methods

### 2.1. Nanostructure Fabrication

**Figure 1:**
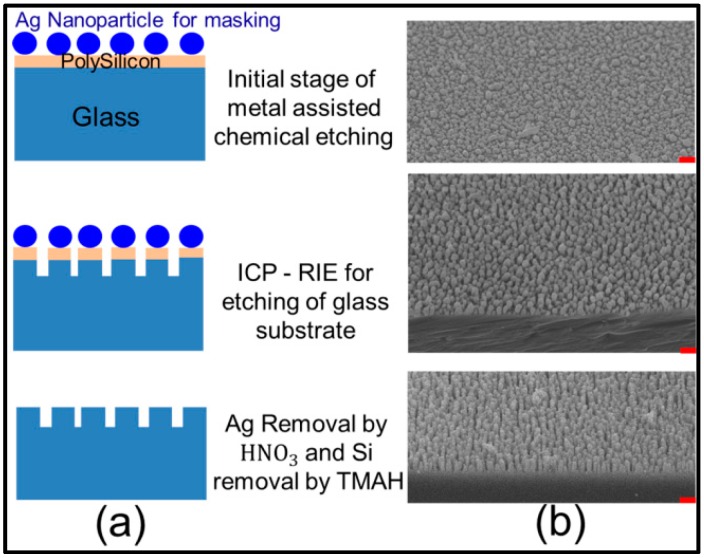
(**a**) Complete process for nanostructure formation; (**b**) SEM image for masking and dry etching process of glass nanostructure (all scale bars represent 500 nm).

In our current study, we used a mass-producible mask fabrication method. This fabrication method is also suitable for double-sided mask preparation in a single step. First 100 nm Si was deposited on a borosilicate glass substrate by low pressure chemical vapor deposition (LPCVD). The first step of the fabrication process involves wet chemical etching, which was used for Ag nanoparticle mask formation without incorporating any photolithography process. When a glass sample with a thin Si film was dipped in a solution of hydrofluoric acid (HF) and silver nitrate (AgNO_3_), Si reacted with HF near the vicinity of the Ag nanoparticles. Ag nanoparticles were deposited using electro-less metal deposition assisted by Ag+ ions in an etchant composed of 4.6 M HF and 0.02 M AgNO_3_ [37]. The Ag nanoparticles deposited by wet chemical etching act as a mask for inductively coupled plasma reactive ion etching (ICP-RIE) in the second step. Inductively coupled plasma was generated by perfluorocyclobutane (C_4_F_8_) and oxygen (O_2_) gas (ratio 4:1), RF power of 100 W, ICP power of 200 W and pressure of 13 mtorr. Very anisotropic etch profile of nanostructure was obtained because of mostly vertical delivery of reactive ions. The nanostructure depth was controlled by the etching time of ICP-RIE. Following the fabrication process, Ag particles were removed using a nitric acid solution at 25 °C and the Si layer was removed using tetramethylammonium hydroxide bath at 85 °C. (Figure 1).

### 2.2. Three-Point Bending Test

Three-point bending (3PB) was performed for measuring the bending strength of plain borosilicate glass and different depth nanostructured borosilicate glass samples. All samples were diced using a sawing machine (Disco DAD 2H/6T) with dimensions of 60 mm × 20 mm × 0.7 mm according to the ASTM 855-08 standard [38]. For the 3PB test, the specimens were placed in the material testing machine (Hung Ta HT-2102A) with a load cell (Hung Ta 8336) and were loaded to fail at a displacement rate of 30 mm/min by the load applicator. The bending strength was then calculated using the following equation [38].

(1) $$\sigma_{br}=\frac{1.5F_{r}L}{wt^{2}}$$

where σ_br_, *F*_r_, *L*, *w*, and *t* are the bending strength, load at rupture, span length, width, and sample thickness, respectively.

### 2.3. Fragmentation Analysis Using High Speed Camera

**Figure 2:**
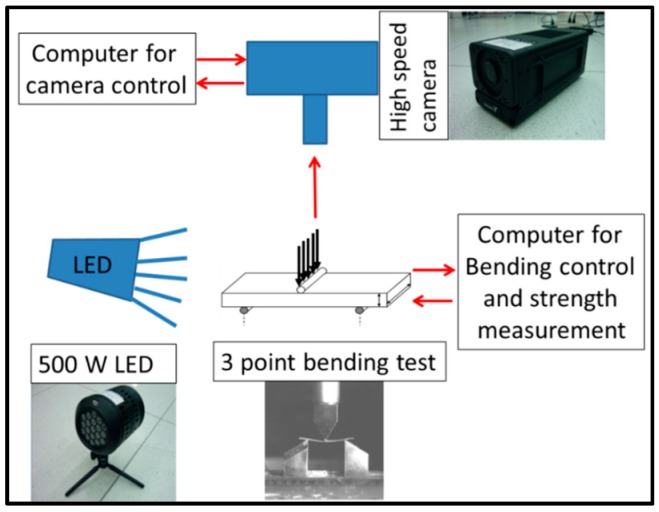
Schematic diagram for fragmentation analysis using three-point bending test.

Fragmentation analysis was conducted for borosilicate glass with dimensions of 60 mm × 20 mm × 0.7 mm using a high-speed camera (IDT Y-4), illuminated by a 500 W halogen lamp. A dynamic response of the 3PB fracture at a frame rate of 2000 frames/s with a 950 μs exposure time and 1280 × 1024 pixel resolution was recorder. The lens (TAMRON A09N) in the camera had a focal length of 30 cm. The schematic of fragmentation analysis by high speed camera set up is shown in Figure 2.

### 2.4. Transparency and Reflection Measurement

We measured both the reflection and transparency for the different nanostructured sample depths using a UV-Vis-NIR spectrophotometer (U4001, Hitachi Inc., Tokyo, Japan) equipped with an integrating sphere for the 400–1000 nm range. The transmittance and reflectance were measured with a fixed incident angle of 5°.

## 3. Results

### 3.1. Bending Strength Measurement for Different Depths of Nanostructure Borosilicate Glass

**Figure 3:**
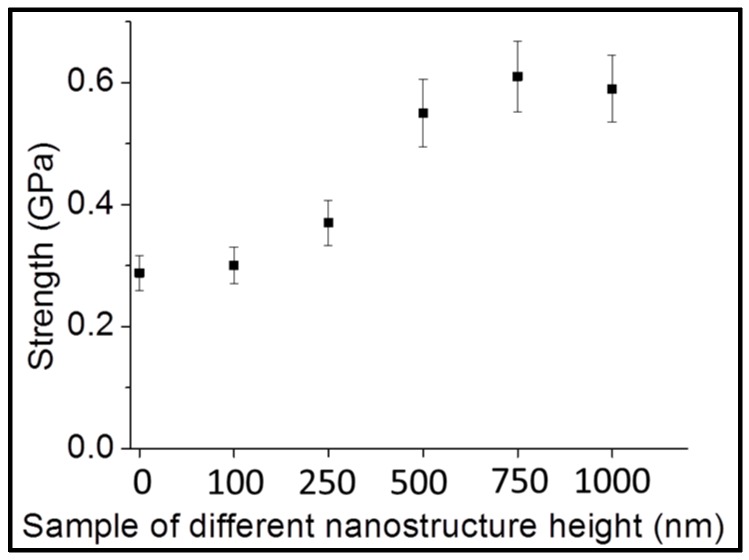
Comparison of bending strengths as determined by 3PB tests between plain (no nanostructure) and different-depth nanostructured borosilicate glass: Bending strength was enhanced from 0.28 GPa for the plain borosilicate glass sample to 0.37 GPa, 0.55 GPa, 0.61 GPa, and 0.59 GPa for 250-nm-, 500-nm-, 750-nm-, and 1000-nm-deep nanostructured borosilicate glass, respectively.

For the borosilicate glass, the bending strength was measured for nanostructured samples with different depths, including 100, 250, 500, 750, and 1000 nm. The bending strength of the plain sample was measured to be 0.28 GPa. After limited improvement for the 100-nm-deep nanostructure, the bending strength improved to 0.37 GPa for 250-nm-deep nanostructured borosilicate glass as shown in Figure 3. The bending strength further increased to 0.55 GPa for the 500-nm-deep nanostructured glass, and to 0.61 GPa for the 750-nm-deep nanostructured glass.

### 3.2. Comparison of Load-Displacement Curve Before and After Nanostructure Fabrication

**Figure 4:**
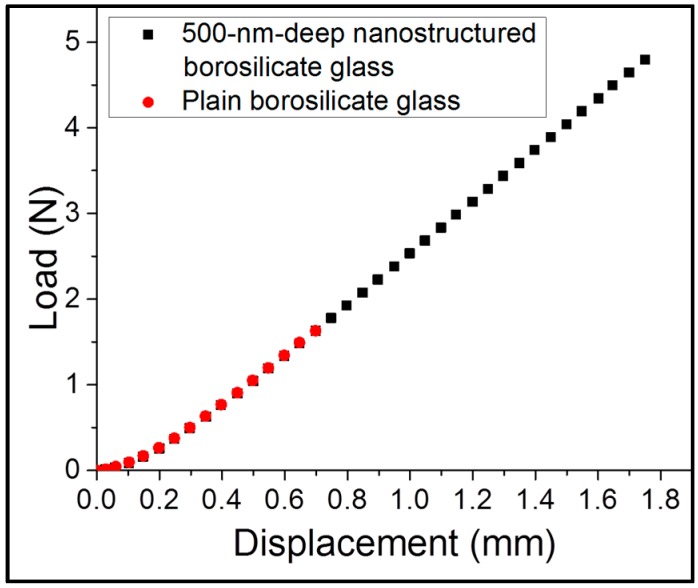
Load-displacement curve for plain borosilicate glass and 500-nm-deep nanostructured borosilicate glass. The almost similar slope for both plain and 500-nm-deep nanostructured confirms unchanged bulk properties after nanostructure fabrication.

The load displacement graph from the 3PB test showed similar responses in the nonlinear and linear regions, as well as in the regions near sudden fracture points. As shown in Figure 4, the stiffness of the nanostructured sample measured by the slope of the load displacement curve, 29.9 kN/m, is highly similar to the stiffness of the plain borosilicate glass sample, 30.1 kN/m.

### 3.3. Fragmentation Analysis Before and After Nanostructure Fabrication

**Figure 5:**
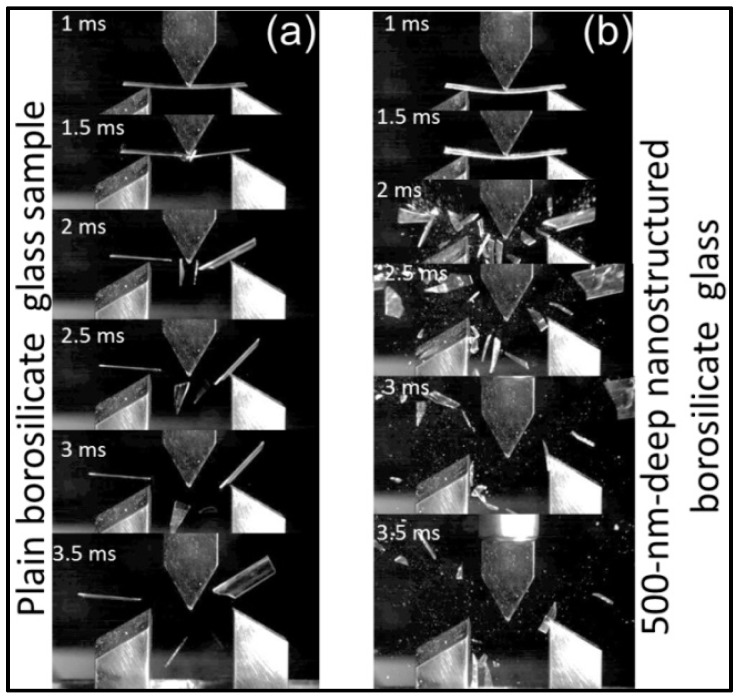
Dynamic fracture behavior captured by a high-speed camera for fragmentation analysis in 3PB test for borosilicate glass with dimensions of 60 mm × 20 mm × 0.7 mm: (**a**) plain borosilicate glass with two major fragments after fracture; (**b**) 500-nm-deep nanostructured borosilicate glass with multiple fragments after fracture.

Fragmentation analysis displayed two major fragments for plain borosilicate glass after failure in the three-point bending test as shown in Figure 5a. The number of fragments multiplied for the 500-nm-deep nanostructured borosilicate glass (Figure 5b).

### 3.4. Transparency and Reflection Comparison for Different Depths of Nanostructured Borosilicate Glass

**Figure 6:**
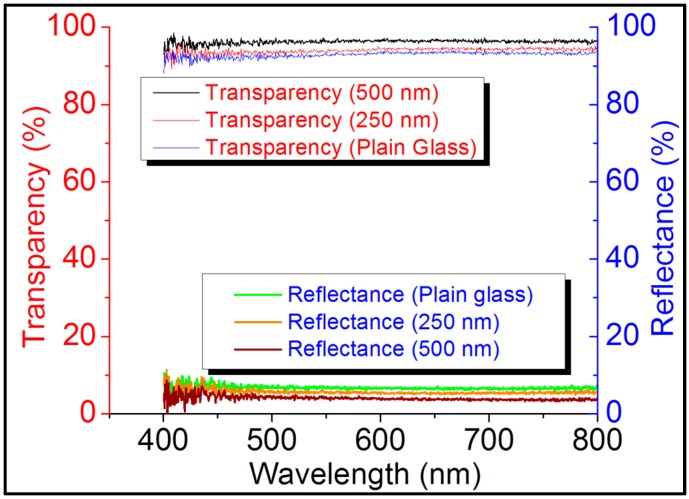
Transparency and reflection measurement in visible light wavelength region (400–800 nm) for plain borosilicate glass, 250-nm-deep, and 500-nm-deep nanostructured borosilicate glass.

Besides bending strength improvement, the total surface reflection from both sides was also reduced from 8% to 6% and then further to 4% for 250 nm and 500 nm nanostructured borosilicate glass samples, respectively, as shown in Figure 6. The transparency improves to 94% and 95% for 250 nm and 500 nm nanostructured borosilicate glass samples, respectively, because of the reduction in surface reflections.

## 4. Discussion

### 4.1. Bending Strength Improvement by Nanostructure Fabrication

Every substrate contains random defects on its surface or subsurface area. Surface and subsurface defects cause local stress enhancement and act as crack initiation points [10]. Nanostructures improve the strength by redistributing local stress at the defect tip to the nearby nanostructured area [25]. More force is required to generate the same stress at the defect tip after stress redistribution, resulting in strength improvement. Since deeper nanostructures are closer to deeper defects, these can redistribute the stress more effectively. Therefore, a deeper nanostructure increases the redistribution of the stress at deeper defects. The bending strength improved from 0.28 GPa to 0.37 GPa for 250 nm-deep nanostructured borosilicate glass as shown in Figure 3 because 250 nm-deep nanostructures are closer to deeper defects compared to plain glass. This explanation was further confirmed by an increase in bending strength to 0.55 GPa for the 500 nm-deep nanostructured glass, and to 0.61 GPa for the 750-nm-deep nanostructured glass. Since defects exist inevitably in all kinds of materials, this nanostructure strengthening method can be applied to all kinds of glass.

### 4.2. Unchanged Bulk Properties After Nanostructure Fabrication

Although almost all strengthening methods affect the bulk properties of a material to increase its strength, nanostructure fabrication has no effect on the bulk properties of the glass substrate. The stiffness and the unchanged Young’s modulus for both plain borosilicate glass and nanostructured borosilicate glass confirm that the bulk properties remained unchanged after the surface nanostructure fabrication (Figure 4).

### 4.3. Higher Strength Confirmation by Fragmentation Analysis

Fragmentation analysis explains the relationship between the number of fragments during fracture and the strength of the material. The higher the number of fragments after the fracture, the larger the sample strength because of the high-strain energy absorption [39]. The presence of two major fragments in the fragmentation analysis results for the plain borosilicate glass sample fracturing illustrates the presence of surface and subsurface defects in plain borosilicate glass (Figure 5a). After nanostructure fabrication, the nanostructures can redistribute the stress near existing defects and cause the sample to break in many small fragments (Figure 5b).

### 4.4. Transparency Improvement by Reduction in Reflection

**Figure 7:**
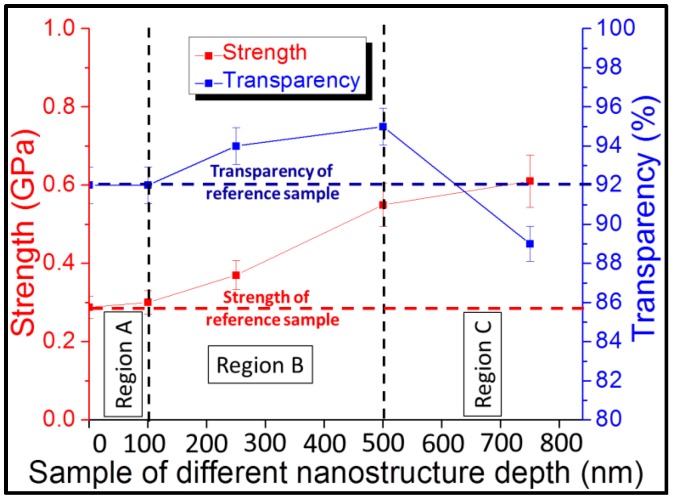
Strengths and transparencies for nanostructured samples with different depths; Region A shows the range of nanostructure depths for which there is almost no change in strength and transparency; Region B is the optimum range for both strength and transparency, where both strength and transparency are improved relative to the plain borosilicate glass; and Region C shows the range of nanostructure depths at which strength is improved and saturated, but transparency is reduced due to scattering.

The glass substrates had refractive indices close to 1.5, which created a surface reflection of approximately 4% from a single side of the plain glass and of approximately 8% from both sides of the glass surface [40,41]. The suppression of reflection over a broad spectral range was achieved by subwavelength texturing at the air-glass boundary, enabling a gradual refractive index transition [42]. For higher-depth nanostructure, the reflection will reduce and transparency will be increased as shown in Figure 6. It is commonly believed that surface reflection will decrease with the increasing nanostructure depth [20] and will increase the optical performance of the glass. However, random nanostructures with a greater depth than the light wavelength can increase the scattering of the light [18,43]. Light scattering increases linearly with the nanostructure depth. Therefore, a reduction in the transparency of 750-nm-deep nanostructured glass to 89% (Figure 7) in our study can be attributed to light scattering by the deeper nanostructure. The loss caused by scattering is negligible for 250 nm-deep nanostructured glass which increased to 1% for 500-nm-deep nanostructured glass and increased further to 9% for 750-nm-deep nanostructured glass.

### 4.5. Optimum Design Window for Enhancement of Strength and Transparency

To determine an optimum range of nanostructure depth, the strength and transparency were measured for samples with nanostructure depths ranging from 0 to 1000 nm. Based on the transparency and bending strength results, we can divide the entire range into three distinct regions as shown in Figure 7. Region A is where the nanostructure depth is less than 100 nm, and random nanostructure fabrication does not affect the strength. Region B is where depths range from 100 to 500 nm, and both strength and transparency were enhanced compared with the reference value. The transparency improved from 92% to 95% at a nanostructure depth of 500 nm, and the bending strength improved from 0.28 to 0.55 GPa. Therefore, to achieve both higher strength and improved transparency, the nanostructure depth should be in the range of 100–500 nm. This range of nanostructure depth is suitable for all applications which utilize both mechanical and optical properties of glass. Optical sensors, displays, panels, and the PV industry are a few of those major applications which can utilize this range of nanostructure depth. Region C is where the nanostructure depth is greater than 500 nm, and the strength increases further to 0.61 GPa for the 750 nm nanostructure sample, but the transparency decreases to 89% because of the scattering in the visible region. Nanostructures with depths greater than 500 nm could improve the mechanical strengths of glass substrates but would degrade their optical performances. Therefore, this region can be useful for applications including pressure sensors and micro-machined mechanical devices which mostly use the mechanical properties of the glass substrate.

## 5. Conclusions

In this study, surface nanostructures fabricated by combined wet and dry etching techniques on borosilicate glass improved the bending strength from 0.28 to 0.55 GPa. Moreover, the nanostructure surfaces exhibited limited transparency enhancements from 92% to 95% as an added advantage. The proposed fabrication process does not require a photolithography mask, which is typically required in the semiconductor industry. Metal-assisted wet chemical etching was used for the silver nanoparticle deposition process, which acted as a mask for dry etching using ICP-RIE. This masking process reduced both the cost and time by depositing a silver nanoparticle mask on both sides of the sample in a single step. The borosilicate glass obtained using this fabrication method exhibited bending strengths enhanced by 96% and improved transparency (from 92% to 95%) in nanostructure depth ranges of 100–500 nm. This nanostructure depth range is suitable for all applications which require both enhanced strength and transparency of the glass substrate. The strength of borosilicate glass can be further improved by increasing the nanostructure depth for applications including pressure sensors and micro-machined mechanical devices where high transparency is not required. Our proposed method can be used to enhance the mechanical and optical performance of all glass substrates without changing their current manufacturing process.

## Abbreviations

The following abbreviations are used in this manuscript:

PV	Photovoltaic
LPCVD	Low pressure chemical vapor deposition
ICP-RIE	Inductively coupled plasma reactive ion etching
3PB	Three-point bending
